# Detangling the Effects of Environmental Filtering and Dispersal Limitation on Aggregated Distributions of Tree and Shrub Species: Life Stage Matters

**DOI:** 10.1371/journal.pone.0156326

**Published:** 2016-05-26

**Authors:** Qing-Song Yang, Guo-Chun Shen, He-Ming Liu, Zhang-Hua Wang, Zun-Ping Ma, Xiao-Feng Fang, Jian Zhang, Xi-Hua Wang

**Affiliations:** 1 School of Ecological and Environmental Science, East China Normal University, Shanghai, China; 2 Tiantong National Station of Forest Ecosystem, Chinese National Ecosystem Observation and Research Network, Ningbo, China; 3 Shanghai Chenshan Plant Science Research Center, Chinese Academy of Sciences / Shanghai Chenshan Botanical Garden, Shanghai, China; Chinese Academy of Forestry, CHINA

## Abstract

The pervasive pattern of aggregated tree distributions in natural communities is commonly explained by the joint effect of two clustering processes: environmental filtering and dispersal limitation, yet little consensus remains on the relative importance of the two clustering processes on tree aggregations. Different life stages of examined species were thought to be one possible explanation of this disagreement, because the effect of environmental filtering and dispersal limitation are expected to increase and decrease with tree life stages, respectively. However, few studies have explicitly tested these expectations. In this study, we evaluated these expectations by three different methods (species-habitat association test based on Poisson Clustering model and spatial point pattern analyses based on Heterogeneous Poisson model and the jointly modeling approach) using 36 species in a 20-ha subtropical forest plot. Our results showed that the percentage of species with significant habitat association increased with life stages, and there were fewer species affected by dispersal limitation in later life stages compared with those in earlier stages. Percentage of variance explained by the environmental filtering and dispersal limitation also increases and decreases with life stages. These results provided a promising alternative explanation on the existing mixed results about the relative importance of the two clustering processes. These findings also highlighted the importance of plant life stages for fully understanding species distributions and species coexistence.

## Introduction

Aggregated distributions of species have widely been documented in various natural communities [1, 2]. Revealing underlying mechanisms of such aggregated pattern is of important for understanding species coexistence [1–4], because the pattern itself can directly affect the fate of individuals, particular sessile plants, by determining how much abiotic resources one can access and how strong biotic interactions one will suffer [5–7]. Many ecological processes thus have been proposed to explain the aggregations. Environmental filtering and dispersal limitation are two of the most widely acknowledged clustering processes for tree distributions [2, 8–12]. The environmental filtering can reduce population density in unfavorable habitats, resulting in clustering distributions in heterogeneous environment [13, 14]. The dispersal limitation can generate similar aggregations of trees by limiting the distributions of seedlings around their parents [10, 15].

The two clustering processes were commonly thought to jointly contribute the aggregations of tree [16, 17], yet there has been little consensus on the relative importance of the environmental filtering and dispersal limitation on tree aggregations [9, 10, 18–21]. Several evidence from tropical [19], neotropical [20] and subtropical forests [21] found that distributions of most of tree species were significantly related to abiotic environmental variables (e.g. elevation of topology and soil nutrients), thus supports the dominant role of environmental filtering on tree aggregations. In contrast, Harms et al. found that 83.6% of species distributed independently with habitat types in a tropical forest plot [9]. Seider and Plotkin [10] demonstrated the extent and scale of conspecific spatial aggregation was correlated with the mode of seed dispersal in same Panama plot, thus supporting dispersal limitation. Valencia et al. [18] found similar results in Amazonian forest in which tree patchiness was not significantly related to topographic variation but was largely due to dispersal limitation of seeds.

These inconsistency of the relative importance of the environmental filtering and dispersal limitation were usually explained by exterior reasons, such as the differences of spatial scales [22, 23] and/or environmental complexity in different studies [11, 24–25], while the immanent difference of trees across different life stages was largely ignored. Since both of the environmental filtering and dispersal limitation may shift their effect on species distributions across life stages, similar mixed results on the relative importance of the two clustering processes could be generated [2, 26].

Specifically, the effect of environmental filtering on tree aggregations may increase with life stages simply because species’ requirement for resources generally increases with their ages and tree size [27–30]. In early life stages of trees, environmental filtering may play a minor role, because the nutrient requirement of seedlings can be partly met by nutrients contained in their own seeds [28]. As dying of individuals in unfavorable habitats, the effect of environmental filtering on tree distribution becomes more evident [13]. While the dispersal limitation contributes to the tree distribution mainly via limiting the dispersion of seeds. Thus the effect of dispersal limitation might be the strongest in the early life stages of trees. In late life stages, other processes, such as abiotic resource mediated self-thinning [31] and soil biota-mediated negative density dependence [32–33], can limit the growth, survival and recruitment of conspecifics, particular in cluster with high density of conspecifics, thus might reduce the importance of dispersal limitation on tree aggregations [3, 34, 35]. In addition, the reduction of the importance of dispersal limitation may uneven among species because the extent and scale of conspecific aggregation are different among species [10].

The possible role of life stage on the inconsistent results of the two clustering processes on tree aggregation has been separately acknowledged by several studies, e.g. [36–39], but no study has examined the importance of the environmental filtering and dispersal limitation simultaneously across several life stages of trees in the same methodology framework in a subtropical evergreen broad-leave forest. This forest supports a unique vegetation that stores a large amount of biomass in the Northern Hemisphere, while other areas of similar latitude (about latitude 25°–35° N) are desert or semi-desert [40]. In this study, we analysed the importance of the two clustering processes in different life stages of trees and shrubs for each species in a 20-ha subtropical forest plot. By combining detailed ground survey data on species distribution, topography and edaphic variables with robust approaches for species-habitat association and spatial point pattern analysis, we examined two expectations: (1) the importance of environmental filtering on tree and shrub distribution increases with life stages, and (2) the importance of dispersal limitation is expected to decrease with life stages of trees and shrubs.

## Materials and Methods

### Study area

This study was carried out in the Tiantong National Forest Park (29°48'N, 121°47'E), Zhejiang Province, in eastern China. This region supports evergreen broad-leaved forests, in which the tree canopy is dominated by species in the families of Fagaceae and Theaceae. Valleys and mountain bases are dominated by deciduous broad-leaved species, mixed with evergreens in the sub-canopy and the understory [41]. The area has a typical monsoon climate with a hot and humid summer and a dry and cold winter [41, 42]. Annual mean temperature is nearly 16.2°C, the warmest month (July) temperature is 28.1°C, and the coldest month (January) temperature is 4.2°C [42]. Average annual precipitation is 1, 374.7 mm that mainly occurs from May to August. The parent soil materials are Mesozoic sediments and acidic intrusive rocks, including quartzite and granite. The soil texture is mainly sandy to silty clay loam, and soil pH ranges from 4.4 to 5.1 [41, 42]. We announce that no specific permissions were required for these locations/activities, and the field studies did not involve endangered or protected species.

### Stem-mapping data

**Fig 1:**
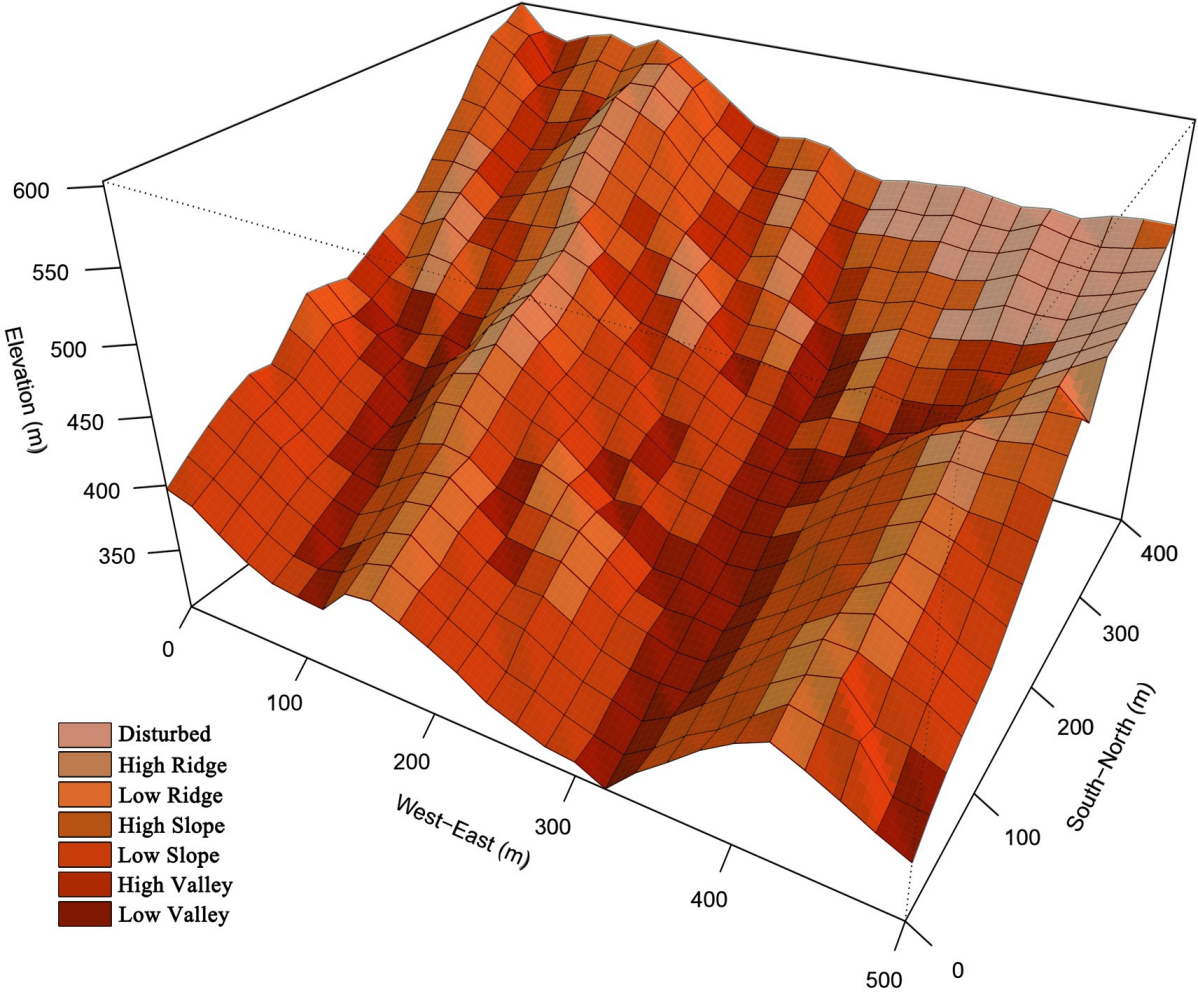
The perspective map of topography and habitat classification of the 20-ha Tiantong forest dynamics plot in eastern China. Each grid is a 20 × 20 m quadrat with color representing different habitat types.

A 20-ha (500 m × 400 m) forest dynamic plot was established within the Tiantong National Forest Park in 2010 (Fig 1). Following the protocols from the CTFS-ForestGEO network [43], all stems of free-standing trees and shrubs with ≥ 1 cm in diameter at breast height (DBH) were tagged, identified, mapped, and measured [44]. A total of 94,605 individual trees and shrubs, belonging to 152 species, 94 genera and 51 families, were recorded in the first census. Tree community in the plot is a typical low-elevation moist evergreen broad-leaved subtropical forest in eastern China.

### Topographic and edaphic variables

A topographic survey was completed on a 20 m × 20 m grid within the 20-ha plot using an Electronic Total Station (Sokkia SET-4120). Three topographic variables, including mean elevation, mean convexity and mean slope, were calculated for each 20 m × 20 m quadrat [9, 18, 45]. These topographic data were used to do habitat classification. Mean elevation ranges from 304.2 m to 602.8 m, with nearly 300 m difference in elevation in the plot. Mean convexity ranges from -5.8 m to 6.9 m, and mean slope varies from 13.8° to 50.3°.

For edaphic variables, 1,292 soil samples covered the whole 20-ha plot at high resolution were collected by both regular and random sampling schemes (S1 Fig). The soil samples were air-dried for more than 30 days, and then were passed through a 0.149 mm sieve. Each sieved soil sample was divided into two subsamples. One subsample of 10 mg was used to analyze total C and total N by elemental analyzer (vario MICRO cube, Elementar, Germany). Another sub-sample of 350 mg was used to analyze total P using flow-injection autoanalyser (SAN++, Skalar, Netherlands). Soil pH of each soil sample was determined by Metterler Toledo pH meter (1:2, H_2_0). For simulating heterogeneous environment in spatial point pattern analysis, standard block kriging was used to obtain three topographic and four soil variables for every 5 m × 5 m quadrat [46, 47].

### Habitat classification

To access species-habitat association, we categorized all 500 20 m × 20 m quadrats into seven habitats (disturbed habitat, high valley, low valley, high ridge, low ridge, high slope, and low slope; see Fig 1 and S1 Table). The north-eastern corner of the plot is a disturbed area where big trees were cut 45 years ago, thus the area was defined as disturbed habitat. For easily comparing with results of previous studies about species-habitat association, e.g. [9, 19, 21], we simply used topographic variables to category quadrats except disturbed habitat in the plot. Previous study in our plot also has found soil variables have strong correlation with topographic variables [48]. Valley, ridge and slope habitats were defined by convexity of quadrats, valley habitat (convexity < -2), slope habitat (-2 ≤ convexity < 2) and ridge habitat (convexity ≥ 2). In our plot, the valley habitat usually has thin and humid soil with strong erosion of water, and interspersed with rocky outcrops, while the ridge habitat has thick and drought soil. The slope habitat is transitional area between ridge habitat and valley habitat. We then chose 450 m above sea level to separate the valley habitat into high valley and low valley, slope habitat into high slope and low slope, ridge habitat into high ridge and low ridge, because large differences in elevation (up to 300 m in this study) can result in changes in vegetation composition and 450 m is the approximate mid-elevation of the plot [9, 49].

### Data analysis

To investigate the effects of environmental filtering and dispersal limitation across tree and shrub life forms, all species in the 20-ha plot were grouped into three growth forms (shrubs, sub-canopy trees, and canopy trees) following the Flora of China [50]. Species in each growth form were divided into three life stages (sapling, juvenile and adult) according to their DBHs (see Table 1 for the classification). To obtain a sufficiently large sample size for point pattern analysis, we selected 36 species with more than 40 individuals and showing aggregated distribution by spatial point pattern analysis under complete spatial randomness (CSR) test in each of three life stages. Aggregated distribution was considered as showing positive deviations of the CSR test within the 0–30 m range by Goodness-of-fit test [51]. To cross verify our predictions that relative contributions of two clustering processes change with life stages, the three following approaches were used, including species-habitat association test, spatial point pattern analysis under the heterogeneous Poisson null model and jointly modeling method.

**Table 1:** Life stage classification of 36 plant species in the 20-ha Tiantong forest dynamic plot.

Growth form	DBH Range (cm)			Richness	Abundance
	Sapling	Juvenile	Adult		
Canopy trees	[1.0, 5.0)	[5.0, 15.0)	≥ 15.0	16	18,681
Sub-canopy trees	[1.0, 2.5)	[2.5, 5.0)	≥ 5.0	14	13,029
Shrubs	[1.0, 1.5)	[1.5, 2.5)	≥ 2.5	6	54,613
Total				36	86,323

#### Species-habitat association test

The effect of environmental filtering on tree/shrub aggregation was detected by the species-habitat association test [52]. Since the mixed effect of the environmental filtering and dispersal limitation may bias the test of environmental filtering, the Poisson clustering point process model, which can model environment-independent aggregated distributions caused by dispersal-like process [2, 20], was used as null model in testing species-habitat association.

Specifically, the first step was to estimate the parameters of Poisson cluster process model based on an observed spatial distribution of each species in each life stage. Spatial range of summary statistics used in parameter estimation was from 0 to 100 m. The parameters contained the information of the number of clumps (kappa), the number of points in each clump (mu) and the range of clump area (sigma) (S2 Fig). A larger kappa indicates a larger number of clumps, and a larger mu indicates a larger number of points in each clump. A larger sigma indicates a larger clump area, and therefore a less clumped distribution. The performance of Poisson cluster model of each species at each life stage were tested by goodness-of-fit method [46, 51, 53], and 88.9% species at sapling stage, 94.4% species at juvenile stage and all species at adult stage were testified that the model reasonably described the pattern of species at the examined scales. The second step was to simulate population distribution for 999 times based on the fitted model in the first step. The last step was to compare the density of observed population with the densities predicted by the fitted model in each habitat [9]. If the observed density of a population is significantly above 99% confidence interval of simulated densities in particular habitat, it means the population significantly associates with the habitat. In order to facilitate comparisons of habitat associations at the three different life stages, we only focused on positive associations [54].

#### Spatial point pattern analysis under the heterogeneous Poisson null model

The effect of dispersal limitation on tree/shrub aggregation was approximately detected by comparing the observed spatial distribution of species and the simulated distributions under the heterogeneous Poisson null model [55, 56], in which the effect of environmental filtering from the observed environmental variables on tree/shrub aggregation was included in the simulated population.

For a given life stage of a species, we considered the following log-linear heterogeneous Poisson model:

$$\text{log}\left(\rho (u)\right)=\mu +H(u)\beta^{T}$$

Where *ρ*(*u*) is a intensity function to describe tree/shrub distribution at any local *u* (e.g. *u* = (*x*,*y*)) in two-dimensional space in our plot. *μ* is an intercept, β is a vector of regression parameters of length *p* and ***H***(*u*) denotes a vector of habitat variables at the spatial location *u*.

The fitted heterogeneous Poisson null model is commonly used to control major effect from environmental filtering in detecting the footprint of dispersal limitation processes on tree/shrub distributions [16, 17]. Any significant clustering found in the observed populations was approximately considered as the effect of dispersal limitation on tree/shrub aggregation.

Specifically, the first step was to precisely estimate the effect of environmental filtering on the observed distribution of species. This step was archived by fitting the Heterogeneous Poisson point process model to the observed distribution for each life stage of each species. Seven topographic and edaphic variables were initially included the model. To reduce the influence of multicollinearity among the environmental variables, we firstly transformed environmental variables into seven independent variables by Principal Component Analysis (PCA). Then a step-wise polynomial regression analysis was used to select significant PCA variables to achieve the ‘best’ heterogeneous Poisson model. Goodness-of-fit tests in S3 Fig showed that the heterogeneous Poisson model was able to control major large scale (e.g. ≥ 50 m) spatial structure of tree species. The second step was to simulate population distribution for 199 times according to the best model found in the first step. The last step is to compare the spatial structure of the observed population with the simulated populations by the inhomogeneous pair correlation function [57, 58]. If the pair correlation function of the observed population of one species is significantly above the 95% confidence interval of the simulated populations between 0–20 m, it means non-habitat clustering processes, mainly dispersal limitation, have detectable effect on the tree/shrub aggregation on that scale.

#### Spatial point pattern analysis under jointly modeling method

Since the above two tests independently examined how environmental filtering or dispersal limitation changes with life stage, we adopted a recently developed method [11] that can evaluate the relative importance of these two clustering processes in the same framework. The ability of this method was archived by jointly modeling environmental filtering and dispersal limitation using a heterogeneous Cox point process model [59] and its associated spatial variance decomposition method [60].

For a given life stage of a species, we considered the following log-linear model:

$$\text{log}\left(\Lambda (u)\right)=\mu +H(u)\beta^{T}+D(u)$$

Where *Λ*(*u*) is a random intensity function to describe tree/shrub distribution at any local *u* (e.g. *u* = (*x*,*y*)) in two-dimensional space in a plot. Given a realization of *Λ*(*u*), the trees/shrubs distribute independently with each other. *μ* is an intercept, β is a vector of regression parameters of length *p* and ***H***(*u*) denotes a vector of habitat variables (aforesaid independent PCA variables transformed from real environmental variables) at the spatial location *u*. The term *D*(*u*) is a random, zero-mean, and spatially correlated “residual effect” modeled by the Matérn covariance function, which can describe different clustering behavior like various dispersal kernels of tree/shrub species. This structure of the model makes it possible to incorporates effect of environmental filtering (the ***H***(*u*)***β***^*T*^ term) and dispersal limitation (the *D*(*u*)) simultaneously, and to evaluate the relative importance of two clustering processes on tree/shrub aggregation.

Given the environmental maps and the observed spatial distribution of individuals in a life stage for each species, parameters of the above Cox process model were estimated by the two-step approach of Waagepetersen and Guan [61]. The model selections (e.g. which habitat variable should be included in ***H***(*u*) and whether there was a significant clustering *D*(*u*) in the model) were conducted by step-wise model reduction and Loosmore’s goodness-of-fit test [51]. More details of parameter estimation, model selection and goodness-of-fit test of Cox model can be found in Shen et al. [11]. Based on the best-fitting Cox point process model for each life stage of each species, the proportion of variance explained by environmental filtering (PVE) and the proportion of variance explained by other clustering processes, such as dispersal limitation, (PVD, note that PVD = 1-PVE) were estimated by the spatial variance decomposition method [11, 60]. To compare the relationship between PVD variation and dispersal syndrome, we further calculated the increment of PVE for each species (the value of later stage minus the value of early stage, such as PVE_juvneile_−PVE_sapling_) in three dispersal groups (gravity-ballistic, animal and wind dispersal based on their fruit anatomy and morphology following Seider & Plotkin [10]).

All analyses were conducted in R 3.1.1 [62]. The analyses for Poisson cluster model and heterogeneous Poisson model were carried out by R Package Spatstat [63], and the analyses for Cox point process model were conducted by the same methods in Shen et al. [11]. The border correction for all the three null model we used was set the option “correction = best” in the R function “envelope” of R package Spatstat [63]. All the R scripts for these analyses were available in S1 File.

## Results

### The importance of environmental filtering across life stages

As we expected, the importance of habitat heterogeneity generally increases with life stages. There was a clear trend that the percentage of species with significantly positive habitat associations increased from 61.1% in the sapling stage to 83.3% in the adult stage. Among the 36 examined species, one third of species were independent of habitat in their sapling stage, but had significantly positive habitat association in their later life stages (S2 Table). Most of the remaining species (22 out of 24 species) were significantly and positively associated with at least one of seven habitat types in one or more life stages (S2 Table). Only 3 species showed a decreasing importance of habitat heterogeneity along life stages (S2 Table).

### The importance of dispersal limitation across life stages

**Fig 2:**
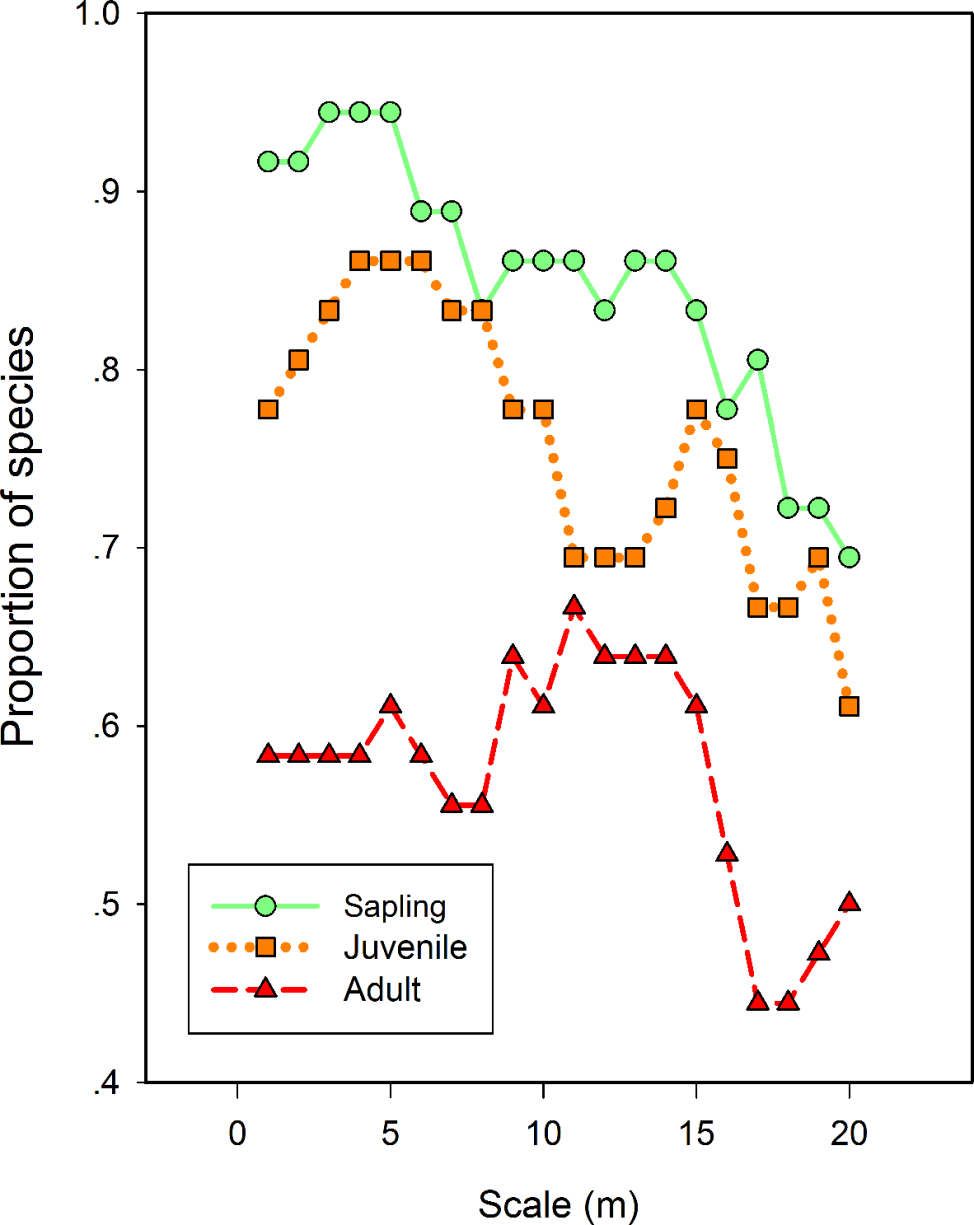
Results of spatial point pattern analysis under heterogeneous Poisson null model. Proportion of species in three life stages showed significant aggregation over different spatial scales.

The percentage of species significantly impacted by dispersal limitation was as high as 94.4% in the sapling stage, and quickly decreased to 88.9% and 75.0% in the juvenile and adult stages, respectively. This decreasing trend held on most of neighborhood scales (e.g. from 0 m to 20 m, Fig 2). In addition, there were 9 species regulated by dispersal limitation in earlier life stages and became independent of dispersal limitation in later life stages (S2 Table).

### Relative contributions of environmental filtering and dispersal limitation along life stages

**Fig 3:**
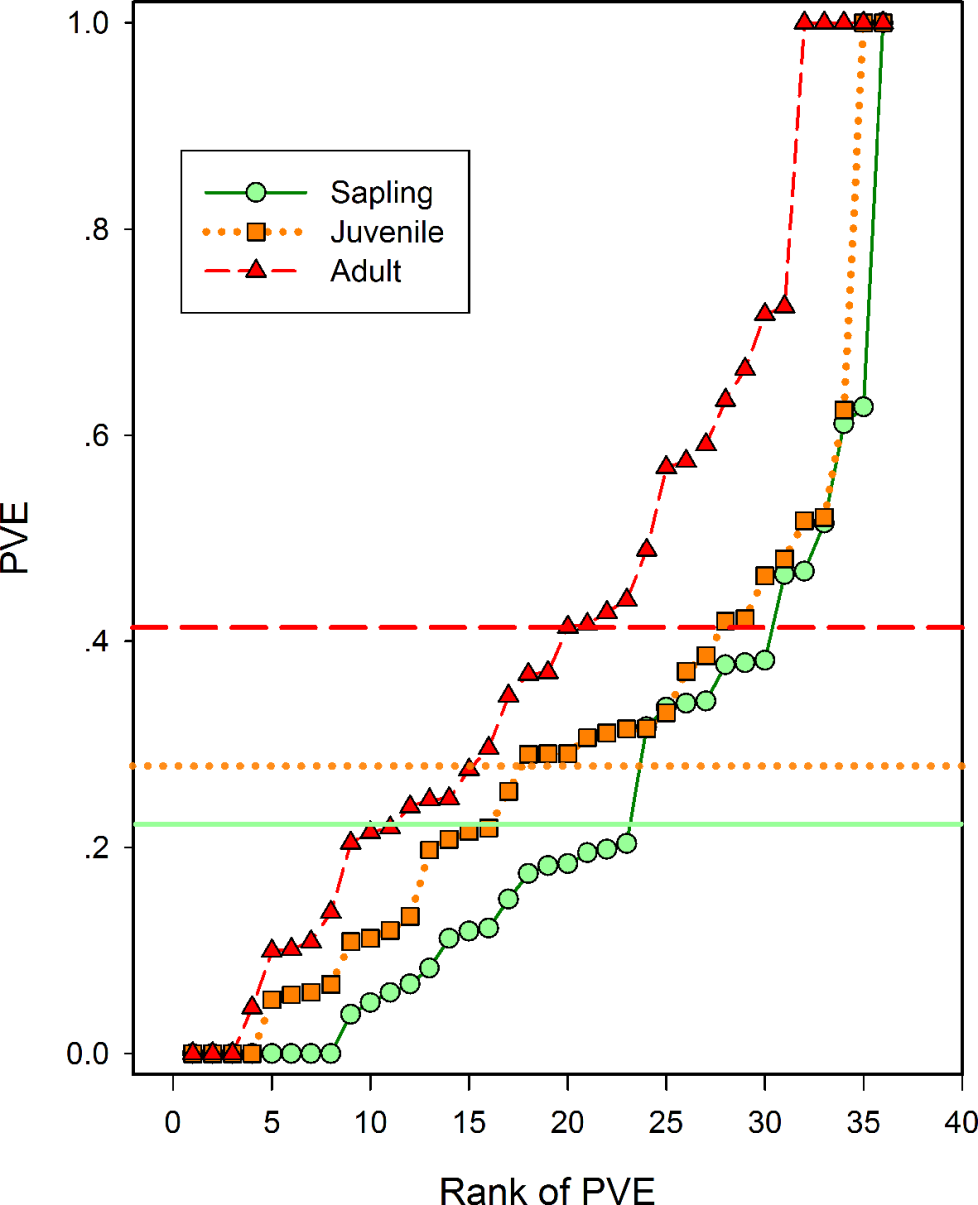
Proportions of variance explained by environmental factors for each species plotted against its rank for species in each life stage by the jointly modeling approach. The solid circle and solid line (green), solid square and dotted line (yellow) and solid triangle and dashed line (red) are mean proportions of variance explained by environmental filtering in the sapling, juvenile and adult life stages, respectively.

**Fig 4:**
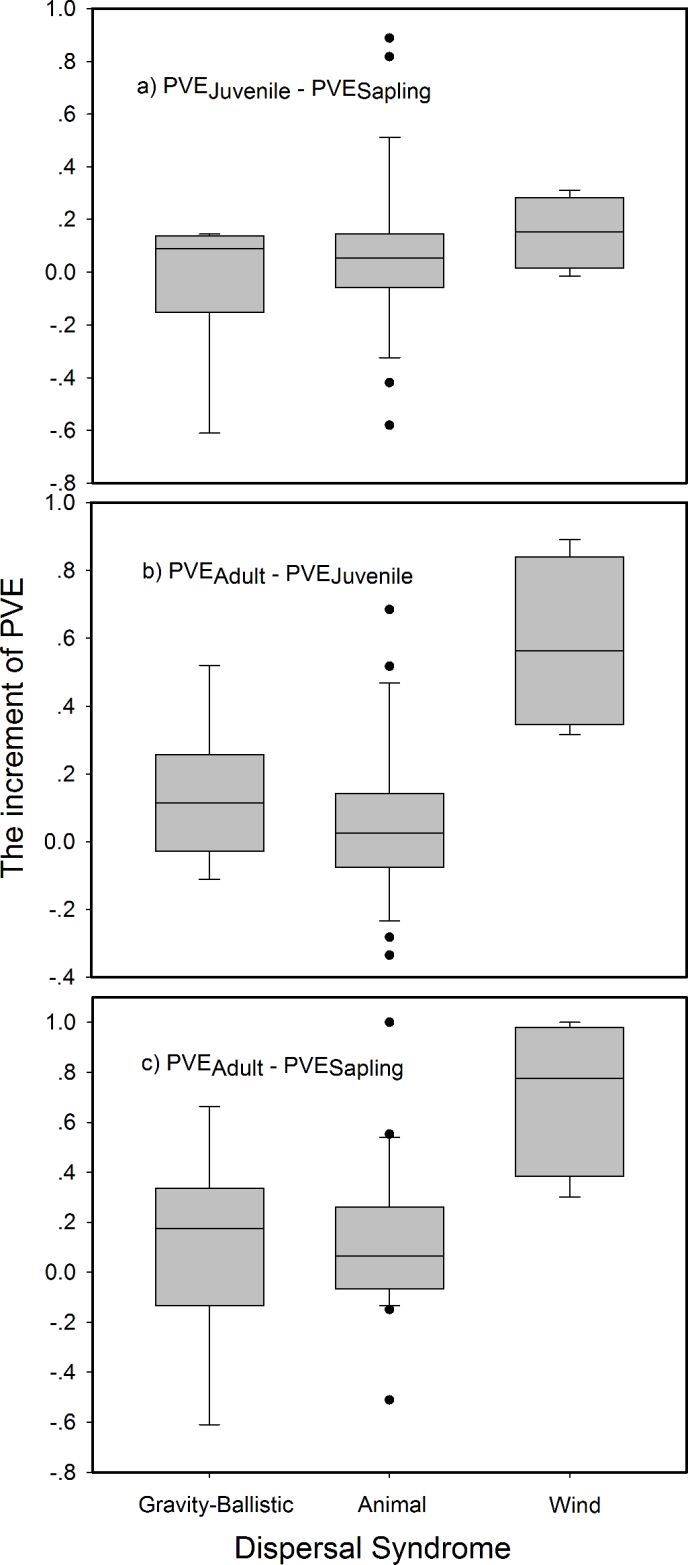
The increment of proportion of variances explained by environmental filtering (PVE) across life stages for different types of dispersal syndrome in the jointly modeling approach.

Using the joint modeling approach, 28 species at sapling stage, 32 species at juvenile and 33 species at adult stage were significantly affected by environmental variables (S3 Table). The percentages of species that were significantly affected by environmental filtering and dispersal limitation (Table 2) were highly consistent with the above results from the previous analyses. The average percentage of variance explained by environmental heterogeneity increased from 0.21 at the sapling stage to 0.41 at the adult stage (Fig 3), suggesting that the importance of environmental heterogeneity increases with life stages. Moreover, the increment of the importance of environmental heterogeneity was significantly greater in species with wind-dispersal mode than other seed dispersal modes, e.g. gravity and ballistic (Fig 4).

**Table 2:** Percentages of species whose spatial distributions were significantly (*P* ≤ 0.05) affected by environmental filtering only, dispersal limitation only, jointly affected by both environmental filtering and dispersal limitation at three tree/shrub life stages and three dispersal modes in the plot by the jointly modeling approach.

Dispersal mode	Life stage	Environmental filtering only (%)	Dispersal limitation only (%)	Both clustering processes (%)
Animal	Sapling	4.0	12.0	84.0
	Juvenile	8.0	8.0	84.0
	Adult	4.0	8.0	88.0
Wind	Sapling	0.0	75.0	25.0
	Juvenile	0.0	0.0	100.0
	Adult	50.0	0.0	50.0
Gravity & Ballistic	Sapling	0.0	28.6	71.4
	Juvenile	0.0	28.6	71.4
	Adult	28.6	14.3	57.1
Total	Sapling	2.8	22.2	75.0
	Juvenile	5.6	11.1	83.3
	Adult	13.9	8.3	77.8

## Discussion

In this study, we tested our predictions about the changes of environmental filtering and dispersal limitation along different tree/shrub life stages in a diverse subtropical forest. Supportive evidence for the predictions arose from a convergence of three different analyses, which consistently showed that the relative importance of environmental filtering and dispersal limitation have opposite changes with life stages from saplings, juvenile to adults. The former increased with life stages and the later decreased with life stages (Figs 3 and 4, S2 Table). This trend of stronger effect of environmental filtering in later life stage is partially related to adults having larger cluster size (e.g. large fitted sigma) and fewer number of clusters (e.g. smaller fitted kappa) in the Poisson Cluster models (S2 Fig). The larger cluster size and fewer number of clusters in the adults compared to juveniles or saplings also hint that environmental filtering might work at a relative larger spatial scale than dispersal limitation does, at least for the examined species in our plot. Results of goodness-of-fit tests on heterogeneous Poisson model in S3 Fig also supported this inference because much more aggregations can be fitted at large spatial scales (e.g. ≥ 50 m).

Our results were consistent with several studies in tropical forests. For instance, Paoli et al. [64] found floristic variation of juvenile sub-community (1–10 cm DBH) was more strongly related to geographical distance (dispersal limitation) than edaphic factors in Indonesia, while the converse held for established trees (≥ 10 cm DBH). Hu et al. [65] found dispersal limitation, which represented by a neighbourhood index, played the most important role in determining the distributions of small trees, and environmental variables played the most important role in determining the distributions of large trees (DBH ≥ 10 cm). For many studies without explicitly considering life stages, the overall effects of environmental filtering and dispersal limitation on tree aggregations has been extensively reported [16–18, 22, 34, 66, 67], but contradictory conclusions about the relative importance of the two types of processes existed for a long time. As examples in introduction, some studies support dispersal limitation [10, 18] and other support environmental filtering [19, 20]. From our analyses, an important reason of these inconsistence may attributed to life stage difference among different communities. Our study provided a promising explanation on the existing mixed results about the relative importance of the two clustering processes.

In addition to the community level of changes in the importance of two clustering processes along life stages, our study also revealed a lot of interspecific differences in the change of the effect of environmental filtering and dispersal limitation across life stages. Notably, there was a significant difference between wind-dispersed and non-wind dispersed (e.g. gravity and ballistic seed dispersal) species on the change of the relative importance of dispersal limitation across life stages (Fig 4). Compared to the gravity-ballistic-dispersed species, more proportion of wind-dispersed species loss effect of dispersal limitation at later life stage (Table 2). From the parameter of Poisson cluster model, the cluster size (sigma) of wind-dispersed species large than animal-dispersed or gravity-ballistic-dispersed species (S4 Fig). Previous study based on data of 187 seed trap in our plot showed similar trend that the seed of wind-dispersed species can be collected at longer distance traps than other species [68]. Species with long dispersal distance may have a large increment of the relative importance of the environmental filtering along the life stages, because species with large dispersal distance always have a less clustered spatial distributions [1, 4], which make it much easier for other processes in the later life stages to reduce the footprint of dispersal limitation. Several other species had no effect of dispersal limitation at later life stages, or affected by dispersal limitation at all three life stages (S2 Table). These interspecific differences can further contribute to the mixed results of community-level importance of environmental filtering and dispersal limitation. Communities with different proportions of wind-dispersed species can easily end up with different relative importance of the two clustering processes on tree aggregation.

It is worth to note that although the importance of the environmental filtering on tree and shrub aggregation is increasing with life stages, the measured environmental data only explained less than 50% of the variation in tree distribution (Fig 4). Frist, we acknowledged that the data accessibility on environmental variables in our study might be result in such relative low importance of environmental filtering, but similar results were also observed in other two forest plots using more comprehensive environmental data [11]. Second, the extent of environmental heterogeneity may affect the relative importance of environmental filtering. For example, our plot and other plots [19, 21] with highly heterogeneous topology showed a higher ratio of species with significant species-habitat association than the BCI plot with a relative flat topology [9]. Therefore, another alternative explanation is that dispersal limitation is really strong, particular in relative homogeneous environment, in formatting the whole spatial structure of tree and shrub species in our plot, even if its importance is decreasing with life stages.

As a summary, we found the importance of the two clustering processes in affecting species distributions varied with life stages in the Tiantong subtropical forest plot. The relative importance of environmental filtering on species aggregation increased with life stages while that relative importance of dispersal limitation decreased with life stages. Such results provided a promising alternative explanation on the existing mixed results about the relative importance of the two clustering processes. It also highlighted the importance of tree and shrubs life stages for fully understanding species distributions and species coexistence. Therefore, it is not quite proper to ask questions like which process, environment filtering or dispersal limitation, is more important for species distributions in a plant community. Better questions about the relative importance of the two clustering processes should explicitly consider the average life stage of species in the plant community, or the particular life stage of the given population. Furthermore, the difference between the two processes along life stages suggests that life stage itself is worth to be explored more closely, and has great potential to be incorporated widely into species coexistence theories in the future.

## Supporting Information

S1 Fig Soil sampling map in the 20-ha Tiantong forest dynamics plot. (TIF)

S2 Fig Boxplot of three parameters of Poisson cluster model (sigma, kappa, mu) shift with life stages. (TIF)

S3 Fig Performance test of the heterogeneous Poisson model in controlling large scale (e.g. ≥ 20 m) structure of species. Almost all species showed significant aggregation above 20 m under the complete spatial random null model (total height of red and blue bar), while these proportions dropped dramatically under the heterogeneous Poisson model (height of blue bar) in all large scales, especially at large spatial scales above 50 m.
(TIF)

S4 Fig Boxplot of cluster size (sigma of Poisson cluster model) of different dispersal syndromes. (TIF)

S1 File R scripts of the analyses in this paper. (TXT)

S1 Table Mean and standard error for topographic (elevation, convexity and slope), floristic (species richness) and structural (density and basal area) characteristics for each 20 × 20-m quadrat among habitats in the 20-ha Tiantong forest dynamics plot. (DOCX)

S2 Table Results of species-habitat association test and spatial pattern analysis (Goodness-of-fit test at 0–20m scale) for 36 species in categories of sapling, juvenile and adult trees in the 20-ha Tiantong Forest Dynamics Plot. (DOCX)

S3 Table Significant associated environmental factors (represented by PCA axis) based on Cox model for each species at different life stages. (DOCX)
