# Supplementary figures and images for: Detangling the Effects of Environmental Filtering and Dispersal Limitation on Aggregated Distributions of Tree and Shrub Species: Life Stage Matters
Source: PLoS One. 2016 May 26;11(5):e0156326. doi: 10.1371/journal.pone.0156326 (PMC4882024; doi:10.1371/journal.pone.0156326)

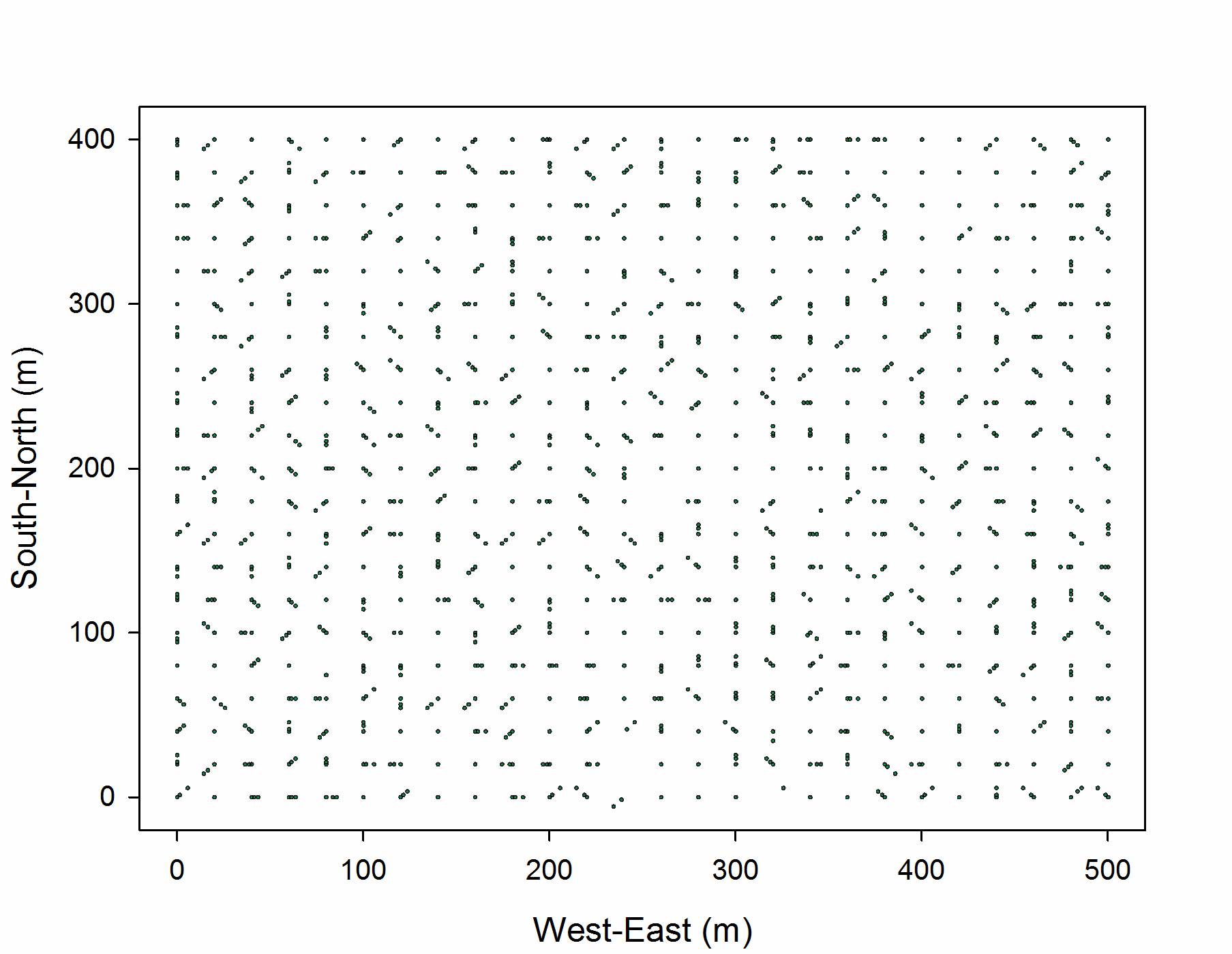

Supplement: S1 Fig — (TIF) [file pone.0156326.s001.tif]

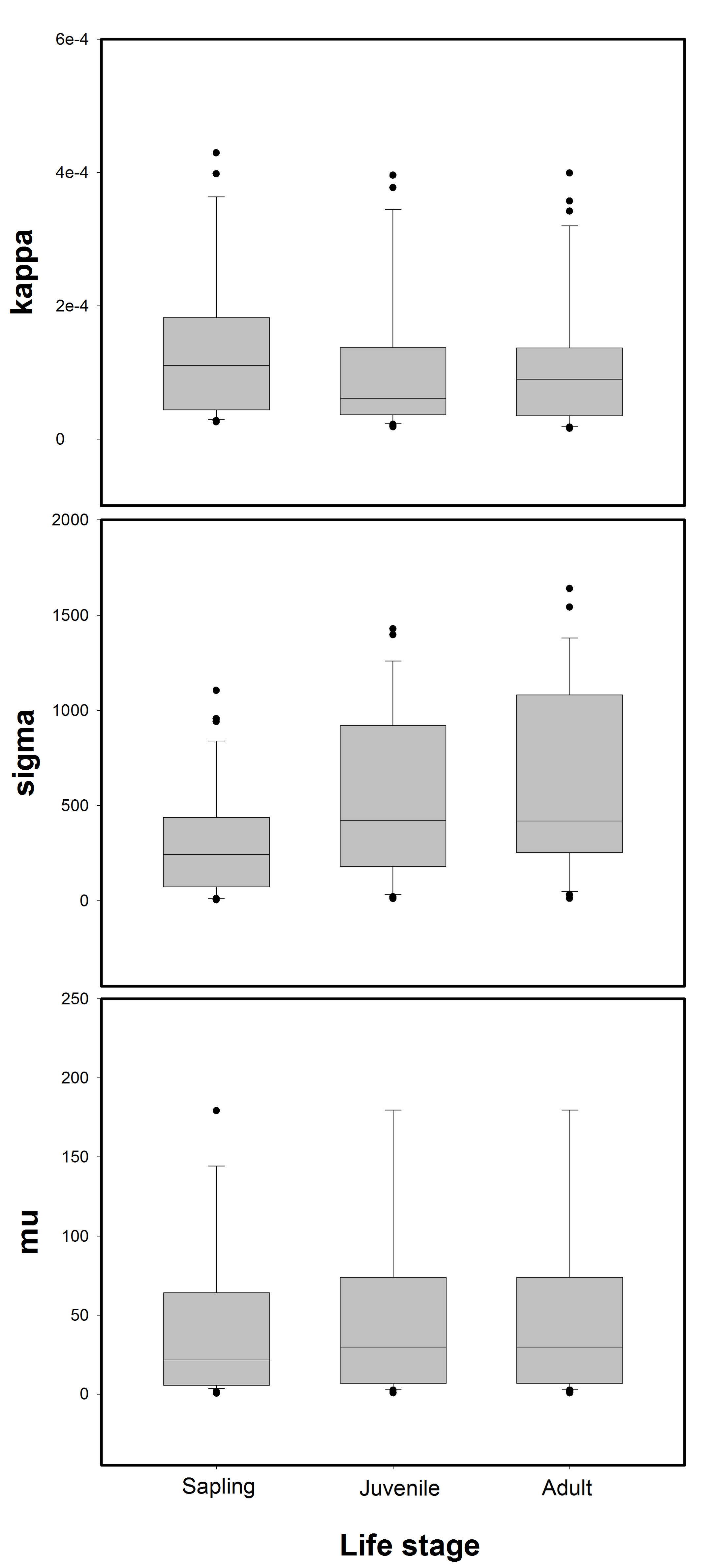

Supplement: S2 Fig — (TIF) [file pone.0156326.s002.tif]

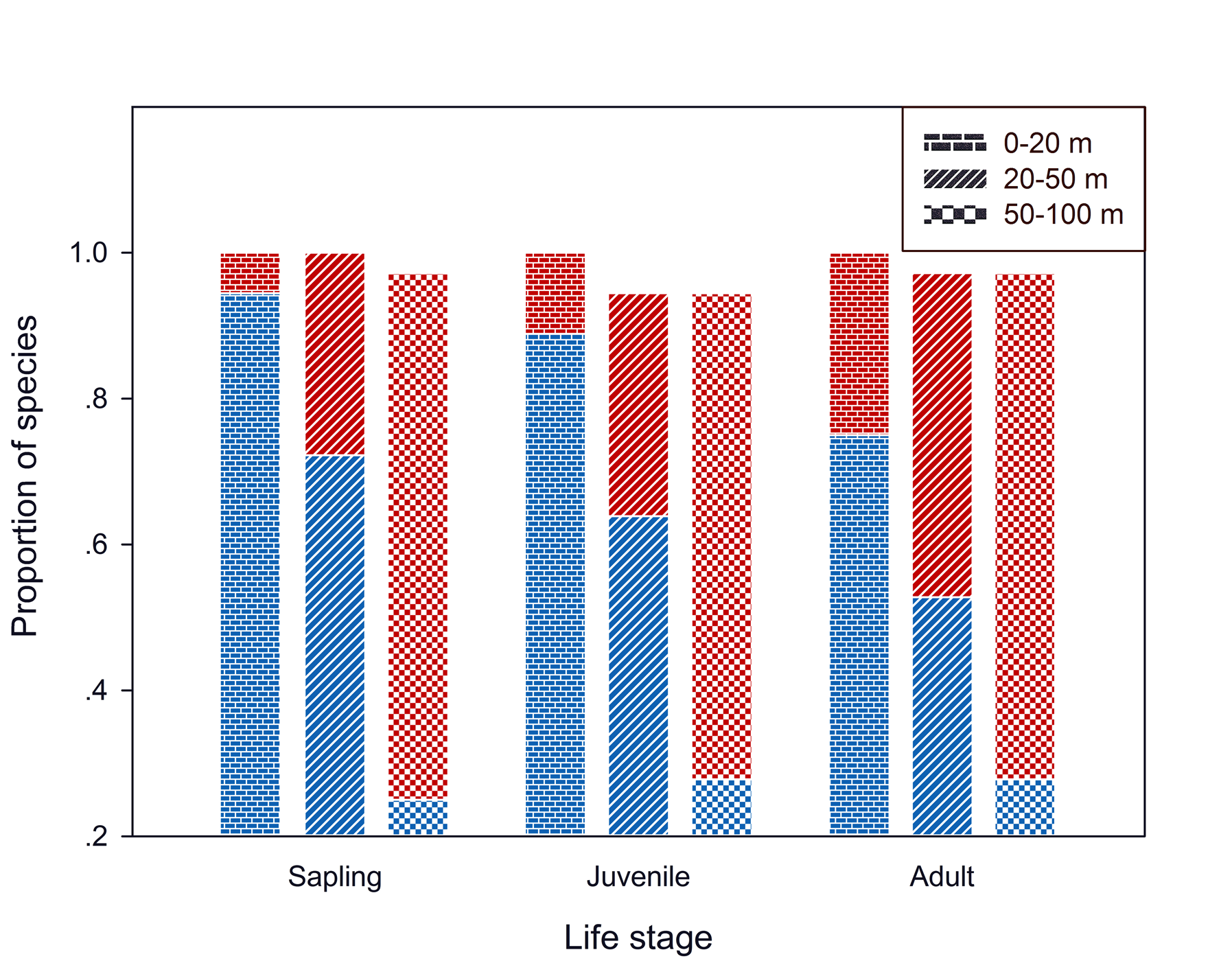

Supplement: S3 Fig — Almost all species showed significant aggregation above 20 m under the complete spatial random null model (total height of red and blue bar), while these proportions dropped dramatically under the heterogeneous Poisson model (height of blue bar) in all large scales, especially at large spatial scales above 50 m. (TIF) [file pone.0156326.s003.tif]

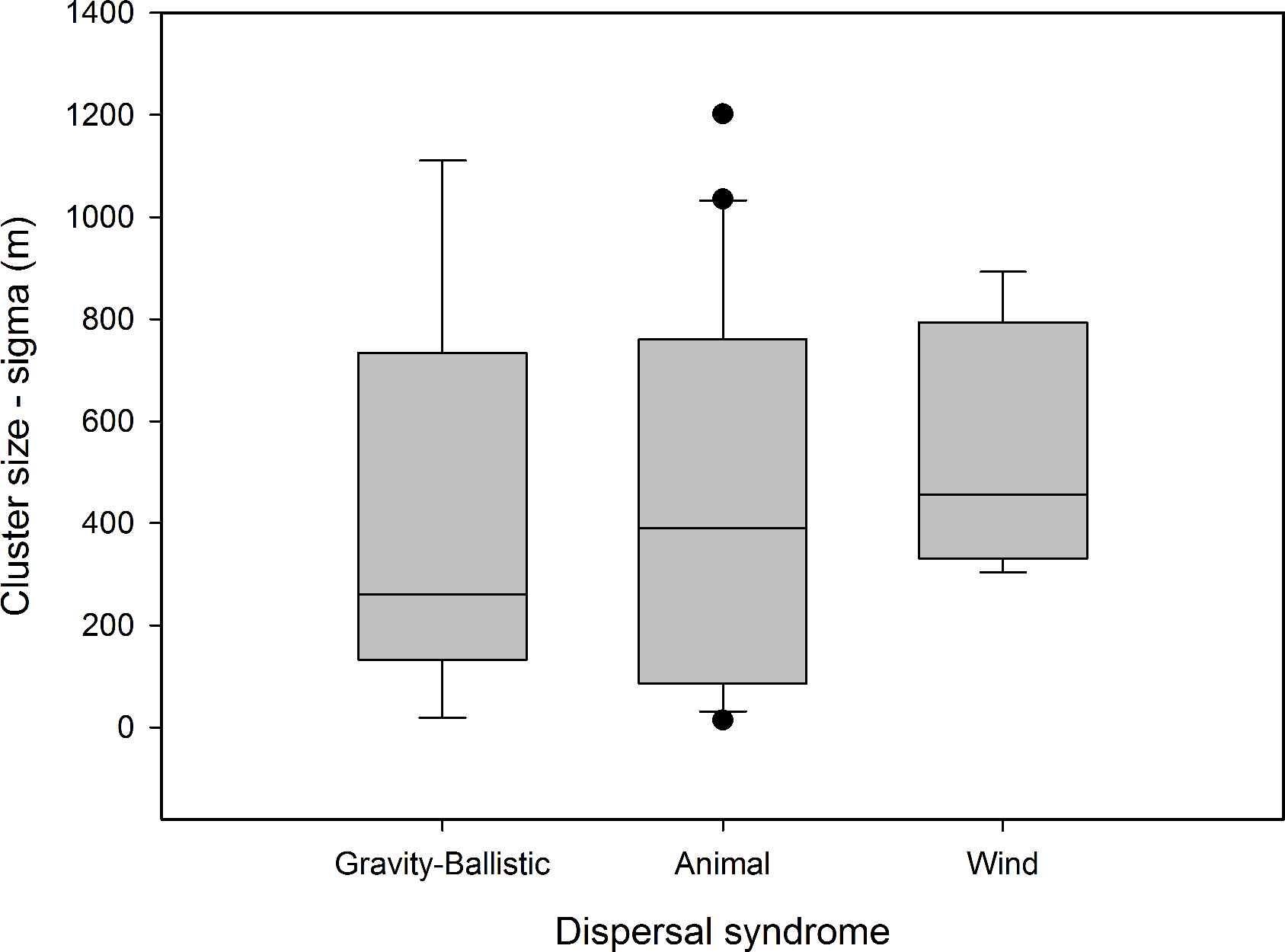

Supplement: S4 Fig — (TIF) [file pone.0156326.s004.tif]
