# Supplementary material for: Detangling the Effects of Environmental Filtering and Dispersal Limitation on Aggregated Distributions of Tree and Shrub Species: Life Stage Matters
Source: PLoS One. 2016 May 26;11(5):e0156326. doi: 10.1371/journal.pone.0156326 (PMC4882024; doi:10.1371/journal.pone.0156326)
Supplement: S1 Table — (DOCX) [file pone.0156326.s006.docx]

**Supporting Information**

**Table S1.** **Mean and standard error for topographic (elevation, convexity and slope), floristic (species richness) and structural (density and basal area) characteristics for each 20 × 20-m quadrat among habitats in the 20-ha Tiantong Forest Dynamics Plot.**

| **Habitat Type*** | **Total area (ha)** | **Elevation (m)** | **Convexity (m)** | **Slope (°)** | **Mean no. of species** | **Mean basal area (m^2^)** | **Mean no. of individuals** |
| --- | --- | --- | --- | --- | --- | --- | --- |
| Disturbed (DIS) | 1.64 | 505.0±12.9 | -0.1±1.3 | 30.1±4.6 | 24.7±6.0 | 1.19±0.32 | 100.0±44.7 |
| Low Valley (LV) | 2.96 | 390.0±36.4 | -3.0±1.4 | 32.5±6.1 | 24.9±5.9 | 1.06±0.27 | 157.2±52.2 |
| High Valley (HV) | 2.00 | 497.4±36.1 | -2.5±1.4 | 36.7±5.0 | 32.8±4.7 | 0.95±0.24 | 180.5±62.0 |
| Low Ridge (LR) | 1.68 | 411.3±23.2 | 3.5±1.4 | 33.8±5.8 | 30.4±4.6 | 1.54±0.26 | 202.2±55.3 |
| High Ridge (HR) | 1.80 | 496.9±35.3 | 3.8±1.5 | 37.1±5.7 | 31.9±4.6 | 1.40±0.27 | 213.7±73.7 |
| Low Slope (LS) | 6.24 | 399.6±32.4 | -0.3±1.3 | 37.0±5.4 | 28.8±6.0 | 1.31±0.34 | 208.7±59.0 |
| High Slope (HS) | 3.68 | 506.4±40.3 | 0.0±1.4 | 38.6±4.8 | 31.4±5.7 | 1.18±0.30 | 208.6±67.9 |

*: Habitat type was defined as: Disturbed habitat where the forest was disturbed in the past; Low Valley (elevation ≤ 450 m, convexity < -2); High Valley (elevation ≥ 450 m, convexity < -2); Low Ridge (elevation ≤ 450 m, convexity ≥ 2); High Ridge (elevation ≥ 450 m, convexity ≥ 2); Low Slope (elevation ≤ 450 m, -2 ≤ convexity < 2); High Slope (elevation ≥ 450 m, -2 ≤ convexity < -2).
