# Supplementary material for: Detangling the Effects of Environmental Filtering and Dispersal Limitation on Aggregated Distributions of Tree and Shrub Species: Life Stage Matters
Source: PLoS One. 2016 May 26;11(5):e0156326. doi: 10.1371/journal.pone.0156326 (PMC4882024; doi:10.1371/journal.pone.0156326)
Supplement: S2 Table — (DOCX) [file pone.0156326.s007.docx]

**Supporting Information**

**Table S2. Results of species-habitat association test and spatial pattern analysis (GOF test at 0-30m scale) for 36 species in categories of sapling, juvenile and adult trees in the 20-ha Tiantong Forest Dynamics Plot.**

| **Species** | **Lifeform** | **Abundance** | | | **Fruit morphology** | **Dispersal Syndrome** | **Habitat association** | | | **Goodness-of-fit test** | | |
| --- | --- | --- | --- | --- | --- | --- | --- | --- | --- | --- | --- | --- |
|  |  | **Sap.*** | **Juv.** | **Adu.** |  |  | **Sap.** | **Juv.** | **Adu.** | **Sap.** | **Juv.** | **Adu.** |
| *Acer pubinerve* | Canopy tree | 195 | 67 | 207 | Winged Nut | Wind | - | - | DIS | Sig.† | - | - |
| *Alniphyllum fortunei* | Canopy tree | 283 | 268 | 82 | Winged Capsule | Wind | HV | HV | LV | Sig. | Sig. | - |
| *Camellia fraterna* | Shrub | 2148 | 3910 | 3221 | Capsule | Ballistic | - | HS | HS | Sig. | Sig. | Sig. |
| *Carpinus viminea* | Canopy tree | 249 | 388 | 238 | Winged Nut | Wind | - | - | HS | Sig. | Sig. | - |
| *Castanopsis carlesii* | Canopy tree | 152 | 92 | 287 | Nut | Animal | LR | LR | LR | Sig. | - | - |
| *Castanopsis fargesii* | Canopy tree | 119 | 85 | 546 | Nut | Gravity | - | - | LR | Sig. | - | Sig. |
| *Choerospondias axillaris* | Canopy tree | 414 | 93 | 845 | Drupe | Animal | HS | - | LS | Sig. | Sig. | Sig. |
| *Cinnamomum subavenium* | Canopy tree | 737 | 330 | 113 | Drupe | Animal | LR | LR,HR | - | Sig. | Sig. | - |
| *Clerodendrum cyrtophyllum* | Shrub | 131 | 204 | 99 | Drupe | Animal | - | - | - | Sig. | Sig. | Sig. |
| *Cleyera japonica* | Sub-Canopy tree | 850 | 459 | 1019 | Berry | Animal | - | - | HV | Sig. | Sig. | Sig. |
| *Cyclobalanopsis nubium* | Canopy tree | 782 | 954 | 748 | Nut | Gravity | - | HS | HR | Sig. | Sig. | Sig. |
| *Daphniphyllum oldhami* | Canopy tree | 116 | 236 | 110 | Drupe | Animal | LR | - | - | Sig. | Sig. | - |
| *Distylium myricoides* | Sub-Canopy tree | 2367 | 1210 | 2721 | Capsule | Ballistic | - | HS | LR | Sig. | Sig. | Sig. |
| *Eurya loquaiana* | Sub-Canopy tree | 11311 | 8010 | 1093 | Berry | Animal | LS | LS | LS | Sig. | Sig. | Sig. |
| *Eurya muricata* | Shrub | 141 | 255 | 291 | Berry | Animal | - | - | - | Sig. | Sig. | Sig. |
| *Eurya rubiginosa* var. *attenuata* | Shrub | 598 | 994 | 388 | Berry | Animal | HR,HS | HS | HS | Sig. | Sig. | Sig. |
| *Ilex buergeri* | Canopy tree | 389 | 189 | 111 | Drupe | Animal | - | HR | HR | Sig. | Sig. | Sig. |
| *Illicium lanceolatum* | Sub-Canopy tree | 112 | 415 | 375 | Drupe | Animal | LV | LV | LV | Sig. | Sig. | Sig. |
| *Lindera rubronervia* | Sub-Canopy tree | 133 | 106 | 101 | Drupe | Animal | - | - | HV | Sig. | - | - |
| *Lithocarpus harlandii* | Canopy tree | 702 | 1093 | 894 | Nut | Gravity | HR,HS | HR,HS | HR,HS | Sig. | Sig. | Sig. |
| *Litsea elongata* | Sub-Canopy tree | 4032 | 2999 | 3364 | Drupe | Animal | LV,LS | LV,LS | LV,LS | Sig. | Sig. | Sig. |
| *Machilus leptophylla* | Canopy tree | 642 | 253 | 283 | Drupe | Animal | LV | LV | LV | Sig. | Sig. | Sig. |
| *Machilus thunbergii* | Canopy tree | 1909 | 673 | 225 | Drupe | Animal | - | HR | DIS | Sig. | Sig. | - |
| *Mallotus apelta* | Shrub | 52 | 76 | 53 | Capsule | Ballistic | HV | HV | LV | - | Sig. | Sig. |
| *Neolitsea aurata* var. *chekiangensis* | Sub-Canopy tree | 1096 | 969 | 1151 | Drupe | Animal | HR | HR | HR | Sig. | Sig. | Sig. |
| *Photinia glabra* | Sub-Canopy tree | 166 | 274 | 255 | Pome | Animal | HR | HR | HR | Sig. | Sig. | - |
| *Rhododendron ovatum* | Sub-Canopy tree | 860 | 1125 | 752 | Capsule | Ballistic | HR,HS | HR,HS | HR | Sig. | Sig. | Sig. |
| *Schima superba* | Canopy tree | 154 | 432 | 652 | Winged Capsule | Wind | HR | HR | LR,HR | Sig. | Sig. | - |
| *Styrax confuses* | Sub-Canopy tree | 164 | 160 | 155 | Berry | Animal | HR | - | - | Sig. | Sig. | Sig. |
| *Symplocos anomala* | Sub-Canopy tree | 1632 | 1230 | 444 | Drupe | Animal | HR,HS | HR,HS | HR | Sig. | Sig. | Sig. |
| *Symplocos lancifolia* | Sub-Canopy tree | 112 | 80 | 167 | Drupe | Animal | - | - | LS | Sig. | - | - |
| *Symplocos laurina* | Canopy tree | 539 | 403 | 87 | Drupe | Animal | - | HV | DIS | Sig. | Sig. | Sig. |
| *Symplocos setchuensis* | Sub-Canopy tree | 524 | 483 | 662 | Drupe | Animal | HS | HS | HS | Sig. | Sig. | Sig. |
| *Symplocos sumuntia* | Sub-Canopy tree | 863 | 489 | 123 | Drupe | Animal | HR | - | - | Sig. | Sig. | - |
| *Syzygium buxifolium* | Shrub | 67 | 167 | 234 | Berry | Animal | HR | HR | HR | - | Sig. | Sig. |
| *Vernicia fordii* | Canopy tree | 62 | 105 | 148 | Drupe | Animal | HV | LS | LS | Sig. | Sig. | - |
| Total |  | 34803 | 29276 | 22244 |  |  | 22 | 24 | 30 | 34 | 32 | 27 |

**Note:** DIS (Disturbed), LV (Low Valley), HV (High Valley), LR (Low Ridge), HR (High Ridge), LS (Low Slope), HS (High Slope) represent seven different habitats. The dispersal syndrome was classified based on fruit morphology following Seidler & Plotkin (2006).

*: Sap. - Sapling; Juv. - Juvenile; and Adu. - Adult

†: Sig. - significant result by Goodness-of-fit test at 0-20m scale
