# Supplementary material for: Detangling the Effects of Environmental Filtering and Dispersal Limitation on Aggregated Distributions of Tree and Shrub Species: Life Stage Matters
Source: PLoS One. 2016 May 26;11(5):e0156326. doi: 10.1371/journal.pone.0156326 (PMC4882024; doi:10.1371/journal.pone.0156326)
Supplement: S3 Table — (DOCX) [file pone.0156326.s008.docx]

**Supporting Information**

**S3 Table. Significant associated environmental factors (represented by PCA axis) based on Cox model for each species at different life stages.**

| **Species** | **Lifeform** | **Significant associated PCA axis** | | |
| --- | --- | --- | --- | --- |
|  |  | **Sapling** | **Juvenile** | **Adult** |
| *Acer pubinerve* | Canopy tree | 4, | 1 | 1,2,3,4,7 |
| *Alniphyllum fortunei* | Canopy tree | None | 3,5 | 1,2,5,7 |
| *Carpinus viminea* | Canopy tree | None | 2,7 | 1,2,4,5 |
| *Castanopsis carlesii* | Canopy tree | 7, | 2,4,7 | 2,4,7 |
| *Castanopsis fargesii* | Canopy tree | None | 2,4 | 2,3,4,5,6,7 |
| *Choerospondias axillaris* | Canopy tree | None | 2,5 | 5 |
| *Cinnamomum subavenium* | Canopy tree | 2, | 1,2,7 | 6,7 |
| *Cyclobalanopsis nubium* | Canopy tree | 1,3,4 | 1,3,4 | 2,3,4 |
| *Daphniphyllum oldhami* | Canopy tree | 2,7 | 2 | 2,5,6,7 |
| *Ilex buergeri* | Canopy tree | 1, | 1,2,4,7 | 2,4,5,7 |
| *Lithocarpus harlandii* | Canopy tree | 1,2,3,4,5 | 1,2,4 | 2,4,5 |
| *Machilus leptophylla* | Canopy tree | 1,2 | 1,2,7 | 1,2,7 |
| *Machilus thunbergii* | Canopy tree | 2,3,4,7 | 1,2,3,4 | 2,7 |
| *Schima superba* | Canopy tree | None | 2,5 | 1,2,3,4,7 |
| *Symplocos laurina* | Canopy tree | 2,4,5,7 | 2,3,4,5,7 | 2,3,4,5,7 |
| *Vernicia fordii* | Canopy tree | None | 3 | 3, |
| *Camellia fraterna* | Shrub | None | None | 4,7 |
| *Clerodendrum cyrtophyllum* | Shrub | 4 | None | None |
| *Eurya muricata* | Shrub | None | None | 7 |
| *Eurya rubiginosa* var. *attenuata* | Shrub | 1,2,5 | 1,2,4,5,7 | 1,4,5 |
| *Mallotus apelta* | Shrub | 3,4,5,7 | None | None |
| *Syzygium buxifolium* | Shrub | 1,2,3,7 | 1,2 | 1,2 |
| *Cleyera japonica* | Sub-Canopy tree | 5, | 5, | None |
| *Distylium myricoides* | Sub-Canopy tree | 3, | 1,2,3 | 1,2,3 |
| *Eurya loquaiana* | Sub-Canopy tree | 1,2,3,5 | 1,2,3,5 | 1,3,5 |
| *Illicium lanceolatum* | Sub-Canopy tree | 1,2,7 | 2,7 | 1,2,7 |
| *Lindera rubronervia* | Sub-Canopy tree | 2 | 2,3,7 | 2,4,7 |
| *Litsea elongata* | Sub-Canopy tree | 1,2,4,5,7 | 1,2,3,4,5,7 | 1,2,3,4,5,7 |
| *Neolitsea aurata* var. *chekiangensis* | Sub-Canopy tree | 2,7 | 2,7 | 2,3,4 |
| *Photinia glabra* | Sub-Canopy tree | 2,4 | 1,2,6 | 1,2,7 |
| *Rhododendron ovatum* | Sub-Canopy tree | 1,2,7 | 1,2,4,5,7 | 1,2,3,4,5,7 |
| *Styrax confuses* | Sub-Canopy tree | 2 | 6, | 2 |
| *Symplocos anomala* | Sub-Canopy tree | 1,2,3,4,7 | 1,2,4,7 | 1,2,3,4,6,7 |
| *Symplocos lancifolia* | Sub-Canopy tree | 4,5 | 4, | 5, |
| *Symplocos setchuensis* | Sub-Canopy tree | 2,5 | 2,4,5 | 1,2,4,5 |
| *Symplocos sumuntia* | Sub-Canopy tree | 2 | 2,4,6 | 1,2,4,7 |

Note: Numbers 1-7 are represented seven axes of PCA (Principal Component Analysis)
